# A fast blind zero-shot denoiser

**DOI:** 10.1038/s42256-022-00547-8

**Published:** 2022-10-31

**Authors:** Jason Lequyer, Reuben Philip, Amit Sharma, Wen-Hsin Hsu, Laurence Pelletier

**Affiliations:** 1 Lunenfeld-Tanenbaum Research Institute, Toronto, Ontario Canada; 2 Department of Molecular Genetics, University of Toronto, Toronto, Ontario Canada

**Keywords:** Image processing, Machine learning, Super-resolution microscopy

## Abstract

Image noise is a common problem in light microscopy. This is particularly true in real-time live-cell imaging applications in which long-term cell viability necessitates low-light conditions. Modern denoisers are typically trained on a representative dataset, sometimes consisting of just unpaired noisy shots. However, when data are acquired in real time to track dynamic cellular processes, it is not always practical nor economical to generate these training sets. Recently, denoisers have emerged that allow us to denoise single images without a training set or knowledge about the underlying noise. But such methods are currently too slow to be integrated into imaging pipelines that require rapid, real-time hardware feedback. Here we present Noise2Fast, which can overcome these limitations. Noise2Fast uses a novel downsampling technique we refer to as ‘chequerboard downsampling’. This allows us to train on a discrete 4-image training set, while convergence can be monitored using the original noisy image. We show that Noise2Fast is faster than all similar methods with only a small drop in accuracy compared to the gold standard. We integrate Noise2Fast into real-time multi-modal imaging applications and demonstrate its broad applicability to diverse imaging and analysis pipelines.

## Abstract (web summary)

Image noise is a common problem in light microscopy, and denoising is a key step in microscopic imaging pipelines. Lequyer et al. propose a self-supervised denoising method and apply it to diverse imaging and analysis pipelines.

## Main

Image noise is the random fluctuation of colour or intensity values that is inherent to image acquisition. It usually presents as a hazy shroud that obscures an otherwise clear visual signal. Image denoising methods try to fix this by removing noise after the fact, usually by exploiting the innate structure and pattern of the underlying signal and leveraging it against the apparent stochasticity of the noise^1^. Denoising is particularly important in live-cell imaging applications, where a balance between the conflicting considerations of resolution, phototoxicity and throughput can force the acceptance of a considerable amount of noise to achieve experimental goals.

Many techniques focus on modelling noise by understanding its origin; for example, confocal microscopy is mainly subject to a combination of Gaussian- and Poisson-distributed noise^2^. However, with deep learning, such explicit models are avoidable by instead training a neural network to learn to map noisy images to their clean counterparts, such as in DnCNN^3^, or even by training it to map noisy pairs of images to one another, such as in Noise2Noise^4^, both are essential components of the CARE toolbox^5^.

However, these methods cannot perform effectively on data that were not well represented in the training set, and hence the training set itself can become a source of bias and variation. Moreover, it is not always practical to acquire representative training data. One example of this is live automated microscopy where real-time image analysis is used to alter the behaviour of the imaging system. Such pipelines are usually confronted with single, noisy images and no additional context. While representative training data could be collected in a separate run, this is time consuming, resource intensive and requires expertise that is not always available. For these reasons, blind zero-shot denoisers have been developed.

Blind zero-shot denoisers train themselves on the very image they are trying to denoise, appealing to no other outside information or knowledge about the distribution and/or variance of the noise in the underlying image. Noise2Void^6^ is one of the earliest methods that can be tailored to achieve this. Noise2Void denoises images by using a masking procedure wherein the neural network learns to fill in pixel gaps in the noisy image. The failure of the network to learn the noise causes it to denoise the underlying image. Although it was trained on entire datasets of images with similar noise levels, Noise2Void can be adapted to denoise single noisy images by restricting the training set to just that image (and virtually every other ‘single-shot denoiser’ including Recorrupted-to-Recorrupted, Noise2Void and Noise2Self, can be adapted in a similar way). The basic idea of Noise2Void was improved and generalized in Noise2Self^7^ and further refined in Self2Self^8^ to achieve single image denoising results that are competitive with traditional fully trained methods. However, all viable blind zero-shot denoisers to date require a considerable amount of time to run (for example, Self2Self takes 4 hours to denoise a single 512×512 confocal image), making them impractical for use in real-time situations.

To alleviate this, we propose Noise2Fast. Our method is inspired by a recently published approach called Neighbor2Neighbor^9^ where the neural network learns a mapping between adjacent pixels. We tune our method to speed by using a discrete four-image training set obtained by an unusual form of downsampling we refer to as ‘chequerboard downsampling’ and train a small neural network on this discrete training set. Although such a method inevitably overfits, we can accurately validate using the original full-sized noisy image since our distorted downsamplings do not locally resemble it, giving us a method that has a natural cut-off time unlike blind-spot-based denoisers. Noise2Fast is faster than all compared methods, and is more accurate than all tested methods except for Self2Self.

Consider a 2D image ${{{\bf{x}}}}\in {{\mathbb{R}}}^{m \times n}$ composed of both signal and noise ${{{\bf{s}}}},{{{\bf{n}}}}\in {{\mathbb{R}}}^{m \times n}$. That is to say

1 $${{{\bf{x}}}}={{{\bf{s}}}}+{{{\bf{n}}}}.$$

Denoising is concerned with the inverse problem of inferring **s** from **x** (or equivalently inferring **n** and then solving for **s**). A neural network attempts to solve this problem by finding a function ${f}_{\theta }:{{\mathbb{R}}}^{m \times n}\to {{\mathbb{R}}}^{m \times n}$ (parameterized by the network weights *θ*) such that

2 $${f}_{\theta }({{{\bf{x}}}})\approx {{{\bf{s}}}}.$$

The most intuitive way to train such a network is by using pairs of noisy/clean images and having the network learn a mapping from one to the other. Noise2Noise trains the network to learn a mapping from different noisy shots of the same image, allowing for training in the absence of clean ground truth data. Specifically, given two noisy realizations of the same underlying signal **s** + **n**_1_ and **s** + **n**_2_ Noise2Noise attempts to learn the mapping

3 $${f}_{\theta }({{{\bf{s}}}}+{{{{\bf{n}}}}}_{{{{\bf{1}}}}})\to {{{\bf{s}}}}+{{{{\bf{n}}}}}_{2}.$$

However, if we assume mean-zero noise and choose a sensible loss function^4^, the network may fail to actually learn the noise **n**_2_, and we will be left with

4 $${f}_{\theta }({{{\bf{s}}}}+{{{{\bf{n}}}}}_{{{{\bf{1}}}}})\approx {{{\bf{s}}}},$$

denoising the image as a result. Although elegant, this method still requires pairs of noisy images to train on.

Recently, interest has grown in methods that can denoise single noisy images, without this added requirement. To fully understand these methods, we need to adopt a different perspective of how neural networks denoise images.

Here we take the view of Krull et al.^6^, based on the concept of receptive fields. The receptive field of a fully convolutional neural network (FCN) is the set of input pixels that were taken into consideration for a given output pixel prediction. For example, in our above scenario suppose $(i,j)\in {{\mathbb{N}}}_{\le m}\times {{\mathbb{N}}}_{\le n}$ are the co-ordinates of some pixel in the output image *f*_*θ*_(**x**). Then the receptive field (RF) of that pixel is the set of indices $\mathrm{RF}(i,j)\subseteq {{\mathbb{N}}}_{\le m}\times {{\mathbb{N}}}_{\le n}$ such that *f*_*θ*_(**x**)_(*i*,*j*)_ depends only upon the value of ${\left.{{{\bf{x}}}}\right|}_{\mathrm{RF}(i,j)}$ (typically this will be a small square patch of the image **x**). We can then view the neural network as a mapping from the input image along some receptive field to its corresponding output pixel, with the goal of finding *θ* such that

5 $${f}_{\theta }\left({\left.{{{\bf{x}}}}\right|}_{\mathrm{RF}(i,j)}\right)\approx {{{\bf{s}}}}\left(i,j\right),$$

for every $(i,j)\in {{\mathbb{N}}}_{\le m}\times {{\mathbb{N}}}_{\le n}$. The question though, is how to train these networks without any actual training data other than the noisy image itself. Blind-spot methods approach this by excluding the centre pixel from the receptive field (either by removing/replacing it^6,7^ or ignoring it altogether using partial convolutions^8^), and training the network to recover this centre pixel from its surroundings. More specifically, they train the network to learn the mapping.

6 $${f}_{\theta }\left({\left.{{{\bf{x}}}}\right|}_{\mathrm{RF}(i,j)\setminus (i,j)}\right)\to \,{{{\bf{x}}}}\left(i,j\right).$$

However, just as in Noise2Noise, the network fails to learn the noise, leaving us with

7 $${f}_{\theta }\left({\left.{{{\bf{x}}}}\right|}_{\mathrm{RF}(i,j)\setminus (i,j)}\right)\approx \,{{{\bf{s}}}}\left(i,j\right).$$

Excluding the centre pixel is crucial and ensures that the network does not just learn the identity. However, a side effect of this is that the neural network does not give proper weight to the pixel itself when computing the output, which is unfortunate, because the pixel itself is always going to be the best individual predictor of its denoised value.

**Fig. 1:**
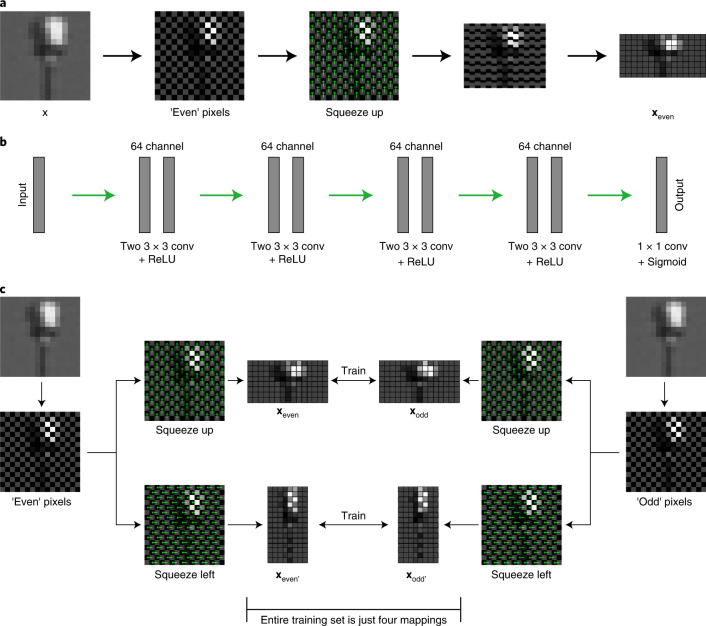
An overview of how Noise2Fast trains itself. **a**, Chequerboard downsampling illustrated. We take our initial image, remove one half of all pixels in a chequerboard pattern, and shift the remaining pixels to fill in the gaps left behind. **b**, Our simple neural network architecture. Inputs can be multi-channel, however, for best results outputs are always single channel (for colour images we predict each channel separately). **c**, Overview of our training scheme. Our neural network learns mappings between pairs of chequerboard downsampled images, each generated from different group of pixels.

Our method takes a related, but slightly different approach. Instead of masking the input image, we explicitly divide the input image in two, by using a simple downsampling method that we refer to as chequerboard downsampling. This process is easier to visualize than explain (see Fig. 1a), however we take our input image **x** and split it into two smaller images composed of the even pixels (where *i* + *j* is even) and odd pixels (where *i* + *j* is odd) respectively, and compress them into the two following $m\times \frac{1}{2}n$ images

8 $${{{{\bf{x}}}}}_{{{{\rm{even}}}}}(i,j)={{{\bf{x}}}}(i,2j+(i\,{{{\rm{mod}}}}\,2)),$$

9 $${{{{\bf{x}}}}}_{{{{\rm{odd}}}}}(i,j)={{{\bf{x}}}}(i,2j+(i\,{{{\rm{mod}}}}\,2)+1).$$

We can call these the ‘up’ chequerboard downsamples, since they involve shifting everything up one pixel to close the image.

Now suppose we train our neural network (Fig. 1b) to learn the mapping

10 $${f}_{\theta }({{{{\bf{x}}}}}_{{{{\rm{even}}}}})\to {{{{\bf{x}}}}}_{{{{\rm{odd}}}}}.$$

We can rewrite this as

11 $${f}_{\theta }({{{{\bf{s}}}}}_{{{{\rm{even}}}}}+{{{{\bf{n}}}}}_{{{{\rm{even}}}}})\to {{{{\bf{s}}}}}_{{{{\rm{even}}}}}+{{{{\bf{n}}}}}_{{{{\rm{odd}}}}}+({{{{\bf{s}}}}}_{{{{\rm{odd}}}}}-{{{{\bf{s}}}}}_{{{{\rm{even}}}}}).$$

Notice that this is analogous to Noise2Noise (equation (3)), except for the addition of the (**s**_odd_ − **s**_even_) term. However, for every $(i,j)\in {{\mathbb{N}}}_{\le m}\times {{\mathbb{N}}}_{\le n}$, we have that **s**_odd_(*i*, *j*) and **s**_even_(*i*, *j*) are adjacent pixels in the original image signal, it is therefore reasonable to think this term would be very small in all but the most highly dynamic regions. Indeed, in our testing, we found that even if we cheat and subtract out the term using known ground truth values, there was no measurable gain in denoising performance. We therefore claim that for most natural images,

12 $${{{{\bf{s}}}}}_{{{{\rm{even}}}}}+{{{{\bf{n}}}}}_{{{{\rm{odd}}}}}+({{{{\bf{s}}}}}_{{{{\rm{odd}}}}}-{{{{\bf{s}}}}}_{{{{\rm{even}}}}})\approx {{{{\bf{s}}}}}_{{{{\rm{even}}}}}+{{{{\bf{n}}}}}_{{{{\rm{odd}}}}}.$$

Then, analogous with Noise2Noise (equations (3) and (4)), training our network as outlined in equation (10) should, in effect, find weights *θ* such that

13 $${f}_{\theta }({{{{\bf{x}}}}}_{{{{\rm{even}}}}})\approx {{{{\bf{s}}}}}_{{{{\rm{even}}}}}.$$

However, in our experiments we have witnessed a much stronger result than this. In particular, we observe that a network trained as in equation (10) will not just learn to denoise the downsampled image, but the entire image as a whole. That is

14 $${f}_{\theta }({{{\bf{x}}}})\approx {{{\bf{s}}}}.$$

To explain this phenomenon, we return to the receptive field-based perspective of equation (6). In this case, our network is trained to learn the mapping

15 $${f}_{\theta }\left({\left.{{{{\bf{x}}}}}_{{{{\rm{even}}}}}\right|}_{\mathrm{RF}(i,j)}\right)\to \,{{{{\bf{x}}}}}_{{{{\rm{odd}}}}}\left(i,j\right).$$

**Extended Data Fig. 1:**
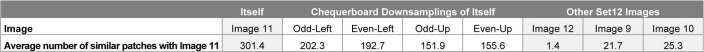
Patch similarity in chequerboard downsamplings. We compare the number of similar patches that exist between Image 11 from Set12, its chequerboard downsamplings and other Set12 images.

It is known, and is often exploited by denoising algorithms, that single images contain significant internal redundancy in the form of recurrent patches^10^. It is also known, and is crucial to some super-resolution methods, that single images have a certain degree of self-similarity, and hence these patches also recur across scales^11–14^. This across-scale patch recurrence implies similarity between the patches in the chequerboard downsampled images and the original full-sized image. We demonstrate this in Extended Data Fig. 1, by comparing the patch-wise similarity between an image and its chequerboard downsamplings. Hence, a neural network trained to learn as in equation (10) may be applicable to the overarching denoising task.

Our method uses this basic principle to generate a small training set of four-image pairs (Fig. 1c). This compact training set allows for rapid network convergence and hence quick single image denoising results that were previously unattainable with such a high degree of accuracy.

## Contribution and significance

**Fig. 2:**
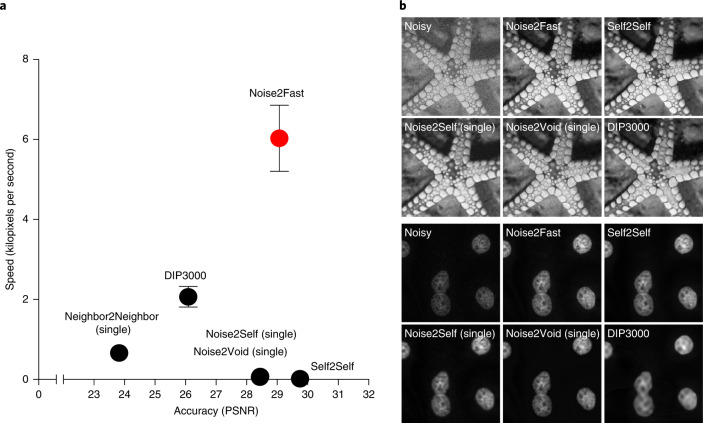
Benchmarking Noise2Fast. **a**, Graph of the speed (in kilopixels per second) of each method on each of the datasets. Error bars represent standard error with *n* = 7. **b**, Visual comparison of each method on starfish image from Set12 and on BPAE cells from Confocal dataset.
- **The first blind single image denoiser that can viably be inserted into live automated microscopy pipelines**. We specifically demonstrate that our method can be seamlessly inserted into ‘smart’ imaging pipeline where the microscope monitors a large field of view containing dozens of cells, detects when a cell enters mitosis, and then zooms in on that cell for the duration of mitosis. Since the window to perform these analyses is only 5 minutes and denoising is just the first step in the pipeline, no other blind zero-shot denoiser could be feasibly placed here.

Our main contributions are as follows:

- **A novel denoising method that combines an unusual downsampling method with Neighbor2Neighbor**. Our method uses chequerboard downsampling to generate a small four-image fixed dataset out of one single image. We then apply our network trained on this smaller dataset to denoise the larger input image, which also serves as our validation set.
- **High accuracy and substantial speed gains over existing methods**. Our method is tailored specifically for speed; using a small four-image dataset ensures rapid convergence, and our validation strategy avoids overfitting. Our method is also quite accurate, in terms of PSNR (peak signal-to-noise ratio) and SSIM (Structural Similarity Index Measure), the only tested method more accurate than the one we propose here is Self2Self, which is an average of 200 times slower (Table 1 and Fig. 2).

**Table 1:** Accuracy and speed of Noise2Fast

Dataset	*σ*	Noise2Self (single)		Noise2Void (single)		DIP3000		Neighbor2Neighbor (single)		Self2Self		Noise2Fast	
		PSNR/SSIM	Time per image	PSNR/SSIM	Time per image	PSNR/SSIM	Time per image	PSNR/SSIM	Time per image	PSNR/SSIM	Time per image	PSNR/SSIM	Time per image
**Set12**	15	30.69/8.71	2,161 s	30.04/8.47	2,682 s	28.51/8.04	70 s	27.97/7.87	198 s	**32.17/8.89**	9,484 s	31.10/8.71	**22** **s**
	25	28.35/7.76	2,161 s	28.41/7.80	2,682 s	26.47/7.07	70 s	26.23/6.87	198 s	**29.88/8.42**	9,484 s	29.05/8.22	**18** **s**
	35	26.59/7.27	2,161 s	27.29/7.71	2,682 s	24.25/5.96	71 s	25.09/6.32	198 s	**28.24/7.99**	9,484 s	27.57/7.81	**19** **s**
	50	25.04/6.87	2,161 s	25.70/7.20	2,682 s	21.19/4.39	70 s	23.43/5.32	198 s	**26.34/7.34**	9,484 s	25.82/7.23	**21** **s**
**BSD68**	25	27.50/7.73	1,619 s	26.66/7.31	2,682 s	25.74/6.85	69 s	25.68/6.92	231 s	**28.70/8.03**	7,962 s	28.12/7.89	**29** **s**
	50	24.53/6.46	1,619 s	24.50/6.05	2,682 s	21.29/4.42	69 s	23.59/5.45	231 s	**25.92/6.99**	7,962 s	25.23/6.70	**26** **s**
**Confocal**	–	36.45/9.31	4,015 s	36.45/9.31	2,682 s	35.16/9.05	102 s	14.78/3.01	400 s	**36.99/9.38**	18,016 s	36.61/9.33	**56** **s**

PSNR and per-image time required to denoise on an RTX 5000 mobile GPU, for each dataset using each method.

## Related work

### Methods that require a training set

The first attempt to apply convolutional neural networks (CNNs) to the task of denoising was in ref. ^15^. This was heavily refined in refs. ^3,16^ (DnCNN) to achieve performance that is still competitive today. Zhang et al. later released FFDNet^17^, a denoising CNN designed with speed in mind which, similar to our method, also uses downsampling, although in a different manner and to an entirely different end (see ref. ^18^).

The main benefit of using trained methods, outside of their outstanding performance, is that they do not require assumptions about the type and structure of the noise, they can simply be trained on noisy/clean pairs of images. However, their reliance on noisy/clean image pairs can be considered a limitation in situations where we do not have access to ground truth images to train on.

To overcome this limitation, Noise2Noise was developed^4^. Noise2Noise can be trained exclusively on pairs of noisy images without any access to ground truth data. It is especially useful in biological imaging where, often, imaging trade-offs dictate that ground truth data cannot ever be obtained.

However, paired noisy images are not always easy to obtain, so there was interest in developing methods that could denoise on unpaired training sets of noisy images from some desired domain. The first method capable of this without having sensitive hyperparameters was Noise2Void^6^. Noise2Void works by training the network to learn a mapping from the noisy image back to itself, masking the centre of each receptive field so as to avoid learning the identity.

This basic model of masking the input is known as a blind-spot network, and was heavily refined and expanded upon in ref. ^19^ and much more recently applied in BP-AIDE^20^ in a manner that is specifically tailored to Gaussian–Poisson noise. In ref. ^21^ they demonstrate a retooled version of BP-AIDE with much faster inference time.

A recently developed alternative to blind-spot networks is Neighbor2Neighbor^9^ which underlies the method we present in this paper. Neighbor2Neighbor learns to map adjacent pixels in the image to one-another, with the idea being that, except in the most highly dynamic regions of the image, adjacent pixels tend to have a similar underlying signal.

Recorrupted-to-Recorrupted^22^ is another recent denoiser. Recorrupted-to-Recorrupted attempts to corrupt single noisy images into noisy image pairs, and then apply a Noise2Noise-like network. Recorrupted-to-Recorrupted is not blind, and requires an estimate of the underlying noise variance and also contains a sensitive ’coefficient of recorruption’ parameter.

Ultimately, all methods listed in this section require a representative training set of noisy images to train on before being applied. In the next section we describe methods that were specifically developed for denoising single noisy images without a training set.

### Zero-shot methods

The first method that directly applied itself to the task of blind zero-shot denoising is Noise2Self^7^. Noise2Self is a very similar method to Noise2Void that achieves slightly better performance, and includes a very thorough mathematical justification for the principles underlying the success of masking-based denoising techniques.

Self2Self^8^ was the first blind zero-shot method whose performance approaches fully trained methods. Self2Self is a blind-spot method, however instead of replacing masked pixels, it ignores them altogether by using partial convolutions^23,24^. Self2Self also introduces the innovative step of adding dropout and averaging across multiple runs of the same image. However, this comes at a high computational cost.

### Non-blind zero-shot methods

BM3D^25^ is one of the gold standards for pure Gaussian noise. It works by unfolding the image into interleaved square patches, clustering those patches based on similarity, and then filtering them before reconstructing the image. BM3D, however, is not blind and takes, as a parameter, an estimate of the standard deviation of the underlying noise. Moreover, BM3D does not work on Poisson noise.

A much more recent learning-based method is Deep Image Prior (DIP)^26^. DIP works by taking a neural network with randomly initialized weights, and training to reconstruct the noisy image. Similar to Noise2Noise, it will fail to learn the underlying noise (at least at first) and instead learn to output the signal. DIP is highly sensitive to the number of iterations, and will quickly overfit if trained too long, for this reason it is not completely practical as a blind denoiser. For our experiments, we force it to be blind by using a fixed iteration number, however, the results it attains are far below what a non-blind version of this algorithm can reach.

**Extended Data Fig. 2:**
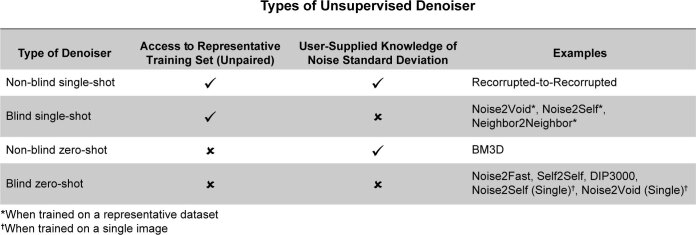
Comparing different types of unsupervised denoiser. We classify the most popular unsupervised denoisers based upon how much information they have access to prior to inference.

Since it is easy to confuse the various different types of unsupervised denoiser, we have included a chart in Extended Data Fig. 2 to clarify the distinctions.

## Results

### Accuracy

The benchmarking of reference datasets was carried out using a single laptop GPU (RTX 5000 mobile GPU) to better approximate the modest (although still powerful) computational capabilities of the average end user. However, because of the massive amount of time required to test Self2Self on 68 images under these constraints, we rely on their previously published accuracy measurement for comparison and estimate time per image using a random sample of five images for this dataset only. On all other datasets (Set12 and Confocal), we run Self2Self on the entire set to obtain accuracy and speed.

On synthetic Gaussian noise our method outperforms everything except Self2Self, which beats us by 0.6–1.0 PSNR across Set12 and BSD68 (Table 1 and Fig. 2). We also tested our method on confocal microscopy images, where again we are slightly less accurate than Self2Self, but outperform everything else. Visual comparison of the results (Fig. 2b) indicate that Noise2Fast appears to smooth the image less than the other methods, creating a more textured look. But overall, every method performed very similarly on the confocal microscopy dataset, except for DIP, which likely needed more iterations to converge, and Neighbor2Neighbor, which seems to not really be suited to zero-shot denoising (nor was it ever intended to be).

**Extended Data Fig. 3:**
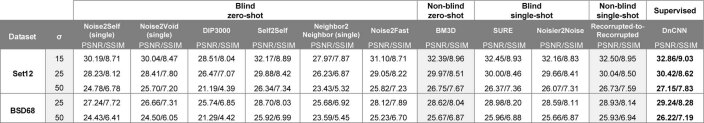
Additional accuracy comparisons on Gaussian noise. Comparing the accuracy performance of Noise2Fast on Gaussian noise, against a myriad of other methods.

For comprehensiveness, we also do pure accuracy comparisons for a myriad of other methods in Extended Data Fig. 3, including Noisier2Noise^27^ and SURE^28,29^ where compared methods have access to varying degrees of additional information ranging from an estimate of the noise level (BM3D) to a full representative noisy/clean dataset (DnCNN).

### Speed

We next sought to determine how fast our method is compared to other algorithms. Our results show that Noise2Fast is considerably faster than all tested methods, an average of 200 times faster than Self2Self the only method that exceeds us in accuracy (Table 1 and Fig. 2).

**Extended Data Fig. 4:**
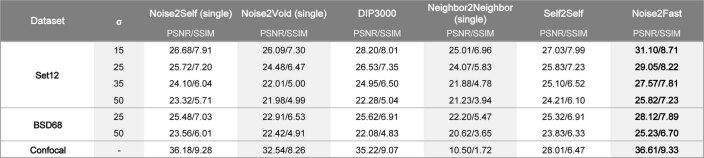
Comparing accuracy when we force methods to be fast. We show the accuracy reached by each method, by the time Noise2Fast has completed its denoising.

Because ’speed’ is just a reflection of the maximum number of iterations we allow each method to run (a parameter we borrow from their published code where possible), we also compared the accuracy of each method if we set the maximum number of iterations so that each program only runs for as long as Noise2Fast takes to fully denoise the image (Extended Data Fig. 4). In this case, it is easy to see that no competitor even approaches the accuracy Noise2Fast can achieve in such a short amount of time.

### Biological data

**Fig. 3:**
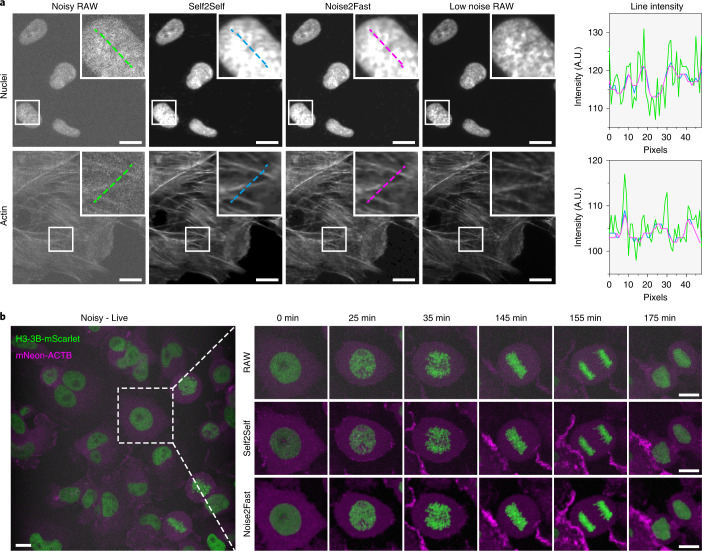
Performance of Noise2Fast on our own microscopy images. **a**, Comparison of Noise2Fast and Self2Self on epifluorescence images of actin and nuclei in RPE-1 cells with corresponding line intensity profiles. **b**, Comparison of live confocal imaging of endogenously tagged nuclei (H3-3B-mScarlet) and actin (mNeon-ACTB) in MDA-MB 231 cells. Scale bars are 10 μm.

Next we determined the speed of Noise2Fast on larger image datasets of both fixed and live cells acquired on our imaging systems. For this, MDA-MB 231 cells were fixed and either stained for actin and DNA or endogenously tagged with H3-3B-mScarlet and mNeon-ACTB (Methods). The performance was compared using two different imaging modalities: epifluorescence for the fixed cells and resonance scanning confocal microscopy for the live cells. Based on the linearity of the intensity measurements of our imaging system, our results indicate that we can achieve relatively clear images while exposing our images to 400-fold less light (Fig. 3). Although Self2Self achieves similar, if not slightly better, results, processing time was significantly longer, more specifically Self2Self required 596 core days versus 0.7 for our method to process the video in Fig. 3b on a Tesla V100.

### Effect on downstream analysis

**Fig. 4:**
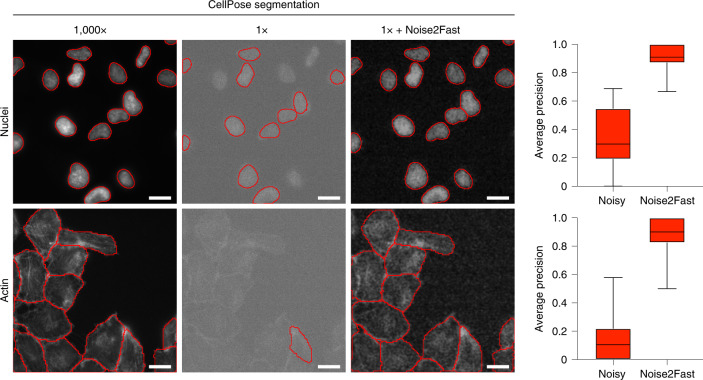
We demonstrate that Noise2Fast significantly improves downstream segmentation with CellPose (a generalist segmentation tool) on our data. We show that by using Noise2Fast on low exposure 1× (0.1 milliseconds) images, we can achieve generalist segmentation results that nearly match that of our high exposure 1,000× (100 milliseconds) pseudo ground truth images. We tested on four crops extracted from each of three fields of view, for a total of eleven images in each dataset (one image was excluded because it contained nothing). All images were taken from the same sample. Scale bars are 10 μm.

We next sought to determine if Noise2Fast improves downstream segmentation tasks. Towards this, in Fig. 4 we show how denoising impacts segmentation results using CellPose^30^. We chose CellPose for this comparison because it is commonly used and it pairs well with Noise2Fast since both are generalist. Our results show a clear improvement in segmentation accuracy when the image is first denoised using Noise2Fast, as quantified using the average precision metric used in refs. ^31,32^. Our results highlight the need to incorporate denoising as a first step in image analysis pipelines when noise levels are high, and in this case we show that Noise2Fast can effectively serve in this role. For a more thorough investigation of how denoising improves segmentation, see ref. ^31^.

### Application

**Fig. 5:**
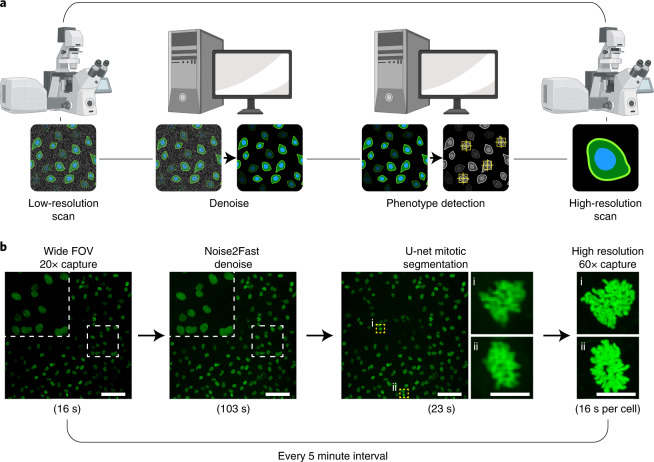
Application of Noise2Fast. **a**, Proposed automated pipeline where the microscope detects a transient phenotype of interest, and both zooms in on and images any cells displaying that phenotype. **b**, Such a pipeline for detecting mitotic cells. Every 5 minutes, the microscope captures a wide field of view 20× image, denoises using Noise2Fast, scans this large field of view for any mitotic cells and then zooms in on them to image at 60×. Under these time constraints, we can detect up to eight mitoses in each 5 minute interval. Scale bars are 100 μm. Created with BioRender.com.

We show that our method can be integrated into time-sensitive automated microscopy pipelines. In particular, we performed an experiment where the microscope monitors a large field of view (FOV) at 20× (containing about 40 cells) over 8 hours (Fig. 5). Whenever the microscope detects a mitotic cell in the large FOV, it zooms in on that cell and images at 60×. Mitotic detection is achieved by first using CellPose to segment the cells, and then distinguishes mitotic from interphase cells by using a cut-off on the standard deviation of the pixel intensity value (anything over 300 is deemed mitotic). This experiment allows us to scrutinize phenotypes that manifest prominently during mitosis, such as centrosome amplification, without sacrificing throughput.

## Conclusion

We presented Noise2Fast, a blind zero-shot denoiser that rapidly converges to accurate results using only the input image to train on. Our key innovation is building a small discrete training set based on chequerboard downsampling that enables our network to quickly converge. We can monitor the progress training using original noisy image as validation. The accuracy of our method surpasses all but one tested blind zero-shot denoising methods, namely Self2Self, however Self2Self takes an average of 200 times longer to run and is therefore impractical in real-time situations, such as in live-cell experiments where the microscope must act upon transient information.

To this end, we successfully integrated Noise2Fast into a live-cell analysis pipeline where the microscope detects and zooms in on any mitotic cells. This example shows that our speed gain is not only an asset, but a necessity to these kinds of analyses. Additionally, from a more theoretical perspective, we believe the observed superiority of chequerboard downsampling over traditional 2×2 downsampling is noteworthy, and the implications this has for full dataset-based denoising methods such as Neighbor2Neighbor might be a worthwhile subject of future research.

## Methods

### Noise2Fast implementation details

Here we outline the specifics of our neural network and training scheme, giving the implementation details of the process outlined earlier. We note that for all zero-shot denoisers we test, noise generation is carried out separately from denoising. That is to say, each method is only exposed to one single noisy instantiation of one single image during training.

For our neural network, we use a simple CNN architecture which we explain briefly here, and illustrate in Fig. 1b. We start by performing two 32 channel 3×3 convolutions with ReLU activation. We repeat this step three more times, each time doubling the number of channels. In the final step, we do 1×1 convolution followed by sigmoid activation.

In our initial testing we found that this much simpler architecture outperformed the classical U-net architecture used in the original Noise2Noise paper^4^. Although the results are not that sensitive to the number of hidden layers, we do find a noticeable, albeit small, drop in performance as we add more to our current model. A possible reason for this is that it causes our network to overfit the data much too quickly. This architecture is similar in its simplicity to DnCNN, one major difference being our lack of batch normalization.

The main novelty of our method is how we train it. Consider a 2D image ${{{\bf{x}}}}\in {{\mathbb{R}}}^{m\times n}$. Recall from the theoretical background that we can divide our image in two by using chequerboard downsampling. By taking the even or odd pixels and squeezing them up to fill in the spaces, as depicted in Fig. 1, we can generate two downsampled $m\times \frac{1}{2}n$ images

16 $${{{{\bf{x}}}}}_{{{{\rm{even}}}}}(i,j)={{{\bf{x}}}}(i,2j+(i\,{{{\rm{mod}}}}\,2)),$$

17 $${{{{\bf{x}}}}}_{{{{\rm{odd}}}}}(i,j)={{{\bf{x}}}}(i,2j+(i\,{{{\rm{mod}}}}\,2)+1).$$

We can call these the ‘up’ chequerboard downsamples. Notice that we can also squeeze the pixels left to generate two $\frac{1}{2}m\times n$ images

18 $${{{\bf{x}}}}^{\prime} (i,j)={{{\bf{x}}}}(2i+(j\,{{{\rm{mod}}}}\,2),j),$$

19 $${{{\bf{x}}}}^{\prime} (i,j)={{{\bf{x}}}}(2i+(j\,{{{\rm{mod}}}}\,2)+1,j).$$

Giving us the ‘left’ chequerboard downsamples. Using these we construct a four-image-pair training set (see Fig. 1c for an overview of our training scheme). We then feed this training data one-by-one into our neural network (batch size = 1). At each iteration we compute the binary cross-entropy (BCE) loss between the target and the output of our neural network, and adjust our weights using the Adam optimizer^33,34^ with learning rate set to 0.001.

When we downsample our image we fundamentally distort the pixel lattice and the relationship between adjacent pixels. For example, in a normal image, suppose each pixel is 1×1 μm. Then the pixel immediately above will be 1 μm away, and the pixel immediately to the right will also be 1 μm away. In the case of a chequerboard downsampled image, for example **x**_even_ in Fig. 1, depending on where you are the relationship might be something like: Up: 2 μm, Left: $\sqrt{2}$ μm. The relationship becomes even more complex as you move further away and the net effect is a serious disruption of the underlying relationship between a pixel and neighbours. We note that this is different from adopting a chequerboard masking scheme and training Noise2Void, where the relationship and adjacency between pixels is preserved. And even Neighbor2Neighbor at least preserves ’ratios’ in the sense that the vertical and horizontal scale are divided by a common number, and these proportions do not vary from pixel to pixel.

This has one important effect: while blind-spot methods based on masking get more and more accurate over time without overfitting because they are not given sufficient information to overfit the data, our method performs more like DIP, where accuracy reaches a maximum very quickly, before it starts to plummet as it ultimately overfits the data, this effect happens particularly quickly on our small four-image training set. However, since our distorted downsampled data looks nothing like the original noisy image at a local level, we observe that if we train our four-image chequerboard downsampled set, while we do inevitably overfit our small training set, the original noisy image is basically unseen data for all intents and purposes. Therefore, we can actually use this image as a validation set.

**Extended Data Fig. 5:**
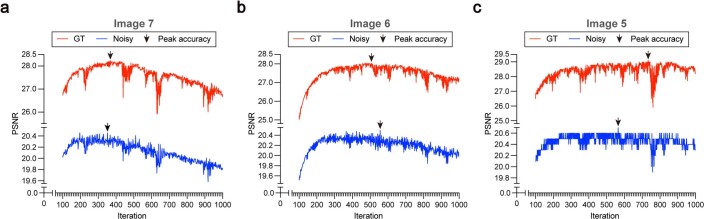
Illustrating our validation strategy on Set12 images. PSNR over time of Noise2Fast on images 5, 6 and 7 from Set12 with *σ* = 25 Gaussian noise. PSNR is calculated by comparing the output of Noise2Fast to both the original ground truth image (red), and to the original noisy image (blue).

More specifically, after each iteration we monitor how our neural network maps our original noisy image **x** to itself, that is how close *f*(**x**) is to the identity mapping on **x**. Our observation is that the optimal time to ‘stop’ training is quite close to the point where the output of the neural network, when applied to original noisy image, most resembles itself. We can validate in this way only because our training set images look so vastly different at a local level than the image we are trying to denoise. In Extended Data Fig. 5 we illustrate how this validation strategy works by comparing over time known ground truth PSNR to the validation PSNR determined by comparing our output to the original noisy image. As can be seen in this figure, the two lines move together and achieve their peaks at roughly the same time. Ultimately, our ability to validate in this way results in a method that converges both quickly and accurately.

### Compared datasets

For blind Gaussian denoising we use the greyscale BSD68^35^ dataset, as was used in ref. ^6^ and a multitude of other denoising papers. BSD68 consists of 68 clear 481×321 photographs to which we add synthetic Gaussian noise. However, to show the effect of spatial resolution on speed and performance, we additionally tested the methods on Set12 which contains a mixture of 256×256 and 512×512 images.

For performance on real-world confocal microscopy, we used a subset of the confocal microscopy images in Fluorescent Microscopy Dataset (FMD)^2^ that we refer to as ‘Confocal’. This dataset contains, among other things, images of biological materials such as cells, zebrafish and mouse brain tissues acquired using commercial confocal microscopes. As described in their paper, ground truth values are estimated by averaging together all 60,000 noisy images in a given set.

### Compared methods

We compare denoising and speed performance against five other blind zero-shot denoisers: Noise2Self^7^, Noise2Void^6^, Self2Self^8^, Neighbor2Neighbor^9^ and DIP^26^. Not all of these methods were originally designed for zero-shot denoising. We will describe how we configured each of these methods in turn, we adhere to published code as much as possible.

#### Self2Self

For Self2Self we use the default published settings of 150,000 iterations and a learning rate of 1 × 10^–4^. We standardize our images differently than Self2Self and some of these other methods. For example, we do not clip our input noisy data [0, 255] at any point. To account for this difference, we have rewritten the dataloaders for Self2Self and other methods to ensure consistency of comparison.

#### Noise2Self

For Noise2Self the only change we make from their published single-shot denoising notebook is to increase the number of iterations from 500 to 20,000, as we found that 500 iterations were not nearly enough to achieve good results on these datasets.

#### Noise2Void

For Noise2Void we found that their ImageJ plugin worked much better than their GitHub code for zero-shot denoising. We therefore used the ImageJ version for benchmarking purposes, which is why our results on this method deviate so much from previous publications. We used a patch size of 64 with 100 epochs and 100 steps per epoch, a batch size of 16 per step, and a neighbourhood radius of 5.

#### DIP

If we fix the maximum number of iterations, DIP becomes a blind denoiser. However, as noted in ref. ^8^, it performs better as a non-blind denoiser. For comparison purposes however, we will set the maximum number of iterations at 3,000, as the authors of DIP have done in their example code on GitHub. This turns it into a blind single-shot denoiser, fully comparable in scope to our method.

#### Neighbor2Neighbor

For Neighbor2Neighbor we used the adaptation of the code found here: https://github.com/neeraj3029/Ne2Ne-Image-Denoising. We adapted the script to zero-shot denoising and attempted in good faith to optimize for the task as best we could, however, we found that the results were inconsistent. We believe that this method is probably best suited to datasets as the authors intended and not single images. We include these results only to illustrate the need to change Neighbor2Neighbor in order to achieve fast and accurate zero-shot denoising results, as we have done in this paper. We do not believe our results are a fair illustration of the power of Neighbor2Neighbor when applied to the tasks it was designed for and we have therefore excluded it from our visual illustrations. We used a learning rate of 0.0003 and trained for 100 epochs, as suggested in their paper for synthetic datasets.

### Fluorescence microscopy images

For fixed immunofluorescence microscopy, RPE-1 cells were fixed with 4% paraformaldehyde at room temperature for 10 min. The cells were then blocked with a blocking buffer (5% BSA and 0.5% Triton X-100 in PBS) for 30 min. Cells were washed with PBS and subsequently incubated with phalloidin-Alexa488 (Molecular Probes) and DAPI in blocking solution for 1 hour. After a final wash with PBS, the coverslips were mounted on glass slides by inverting them onto mounting solution (ProLong Gold antifade; Molecular Probes). For the fixed imaging in Fig. 3a, single Z slices of cells were imaged using Nikon Ti2E/CREST X-Light V2 LFOV25 spinning disk confocal microscope in widefield mode using a 60×/1.4 NA oil-immersion Plan-Apochromat lambda objective. The microscope was outfitted with a Photometrics Prime95B 25 mm FOV ultra-high sensitivity sCMOS camera and images were captured with no binning using the full 25 mm diagonal FOV area at 1,608 px by 1,608 px with a bit depth of 16 bit. After capture, 500 px by 500 px areas were cropped and used as our input dataset. For live imaging in Fig. 3b, endogenously tagged MDA-MB 231 cells were seeded in Nunc Lab-Tek Chamber Slides and imaged on the Nikon Ti2E/AIR-HD25 scanning confocal microscope with temperature and CO_2_ control, using a 40×/1.15 NA water-immersion objective Apochromat lambda S objective. High-speed image acquisition was carried out with the resonance scan head with 2× averaging at 1,024 px by 1,024 px. Full volumes of cells were captured (*Z* total = 20 μm, *Z* interval = 0.5 μm) every 5 minutes for 24 hours. For Fig. 4, single *Z* slices of cells were imaged using Nikon Ti2E/CREST X-Light V2 LFOV25 spinning disk confocal microscope in widefield mode using a 60×/1.4 NA oil-immersion Plan-Apochromat lambda objective. The microscope was outfitted with a Photometrics Prime95B 25 mm FOV ultra-high sensitivity sCMOS camera and images were captured at two different exposures (0.1 and 100 ms) with no binning using the full 25 mm diagonal FOV area at 1,608 px by 1,608 px with a bit depth of 16 bit. After capture, 500 px by 500 px areas were cropped (this you specify to your cropping in this figure) and used as our input dataset. Images were denoised as individual *Z*-slices and max projected. All are displayed with auto scaled LUTs.

### Ablation study

**Extended Data Fig. 6:**
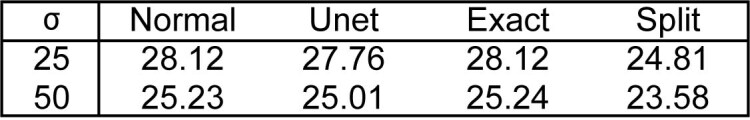
Comparing refinements to Noise2Fast. Comparing different possible refinements to Noise2Fast on BSD68 with *σ* = 25 Gaussian noise.

For our ablation study, we compare three different refinements of the model. First, we replace our simple neural network with a U-net architecture, which is the standard network used in Self2Self and Noise2Void. Again, our performance drops (Extended Data Fig. 6, U-net). Also, using known ground truth values, we manually subtract out the **s**_odd_ − **s**_even_ term in equation (12) and show that this has virtually no impact on our denoising results, hence this term is not having a significant impact on our algorithm (Extended Data Fig. 6, Exact). Finally, we test how well Noise2Fast works if instead of applying it to the original noisy image, we apply it to the chequerboard downsampled images and reassemble them into the full-sized image (Extended Data Fig. 6, Split).

**Extended Data Fig. 7:**
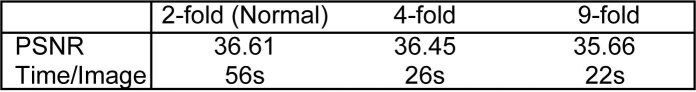
Comparing alternative downsampling strategies. Comparing alternatives to chequerboard downsampling on the Confocal dataset.

We also investigate what happens if we replace our unusual chequerboard downsampling with a more conventional downsample where we divide our image into 2×2 blocks, as used in Neighbor2Neighbor and also 3×3 blocks for fourfold and ninefold downsampling, respectively. This has the advantage making our training set consist of even smaller images to further reduce computation time. We test this on the confocal dataset, and as can be seen in Extended Data Fig. 7, fourfold downsampling doubles speed at only a small drop in accuracy, making this perhaps an attractive solution to those looking for even more speed. Ninefold sampling on the other hand only increases speed marginally and with a much steeper drop in accuracy. The diminishing returns in speed gain are likely a result of the neural network requiring longer to converge when there is less training data per iteration.

**Extended Data Fig. 8:**
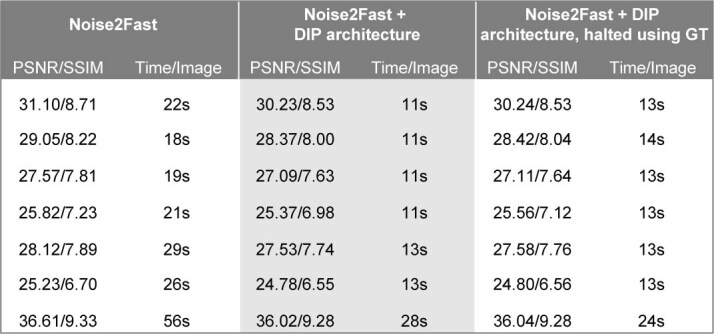
Performance of Noise2Fast with an hourglass architecture. Performance of Noise2Fast using the same architecture as DIP architecture, both validated using the original noisy image and validated using ground truth image.

**Extended Data Fig. 9:**
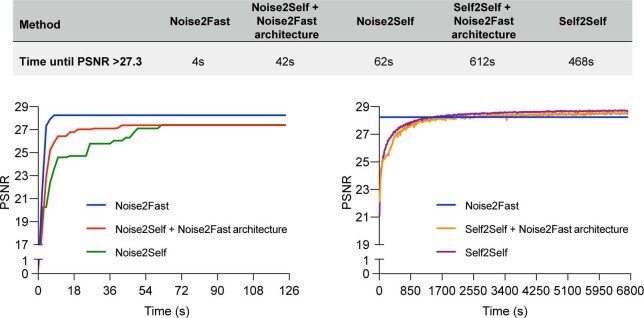
Performance of other methods using Noise2Fast Architecture. Performance over time of Self2Self and Noise2Self on the parrot image from Set12 with *σ* = 25 Gaussian noise, using both their standard architectures and the Noise2Fast architecture.

We also compare the effect our small architecture has on our results by adding the DIP hourglass architecture to Noise2Fast (Extended Data Fig. 8), and also by inserting our architecture into Self2Self (Extended Data Fig. 9) and running it on the parrot image from Set12.

### Source of cell lines

MDA-MB 231 cell line was a gift from R. S. Kerbel (Sunnybrook Health Sciences Centre, Toronto, Canada) and cultured at 37 °C in a humidified environment containing 5% CO_2_. MDA-MB 231 cells were grown in Roswell Park Memorial Institute (RPMI) 1640 medium (Life Technologies) supplemented with 10% fetal bovine serum (FBS). RPE-1 (CRL-4000) cell line was acquired from the American Type Culture Collection (ATCC) and grown in Dulbecco’s Modified Eagle Medium/Nutrient Mixture F12 (DMEM/F12 1:1; Life Technologies) supplemented with 10% FBS.

To generate our endogenous fluorescent cell lines, CRISPR-Cas9 was paired with a repair construct to insert sequences encoding fluorescent proteins via homology-directed recombination into the N- and C-terminus of ACTB and H3-3B, respectively. Briefly, sgRNAs targeting the N-terminus of ACTB (GCCGTTGTCGACGACGAGCGCGG) and the C-terminus of H3-3B (CAGTTGGCTCGCCGGATACGGGG) were cloned into a pX330 plasmid (Addgene plasmid #42230) following the Zhang protocol^36^. To generate the repair constructs, 10,00 bp of homologous genomic sequence surrounding the sgRNA cut site of ACTB and H3-3B was amplified from the genome of RPE-1 cells and subsequently Gibson (M5510AA; NEB) assembled to flank a cassette containing mNeon or mScarlet followed by a 2A peptide into a puromycin or blasticidin resistance gene. Cells were co-transfected with a sgRNA-cloned pX330 plasmid and its matching repair construct using Lipofectamin3000 (Invitrogen) at a 1:1 ratio. An editing period of 72 hours was allotted prior to selection with puromycin (2 mg ml^–1^) or blasticidin (10 mg ml^–1^) to cull non-integrated cells. Knock-in positive cells were subsequently FACS sorted and inspected via fluorescence microscopy.

### Reporting summary

Further information on research design is available in the Nature Research Reporting Summary linked to this article.

## Supplementary information

Reporting Summary

## Data Availability

Benchmarking datasets along with code and reproducibility instructions for Fig. 2 are available on our GitHub (https://github.com/pelletierlab/Noise2Fast). Note that all speed benchmarks were performed on an RTX 5000 mobile GPU, and therefore results may vary according to GPU used. Source input and output images used to make the graphs in Fig. 4 are publicly available on our GitHub (Noise2Fast/Fig5Data). Source data for Fig. 3 is available on our GitHub as well (Noise2Fast/livecells). Figure 1 is a conceptual illustration and does not make use of any datasets, however the image we use to illustrate chequerboard downsampling is a crop of an image available on our GitHub (Noise2Fast/BSD68/19.tif). The minimum dataset for the experiment illustrated in Fig. 5 is publicly available on our GitHub (Noise2Fast/Fig6Data) and the full source data is available on Zenodo (10.5281/zenodo.6949784).

## Code availability

Our code, along with reproducibility instructions, is publicly available at https://github.com/pelletierlab/Noise2Fast. It is also available on codeocean, 10.24433/CO.7967850.v1^37^.
