# Supplementary material for: A fast blind zero-shot denoiser
Source: Nat Mach Intell. 2022 Oct 31;4(11):953–63. doi: 10.1038/s42256-022-00547-8 (PMC9674521; doi:10.1038/s42256-022-00547-8)
Supplement: Supplementary file 1 — Reporting Summary [file 42256_2022_547_MOESM1_ESM.pdf]

nature research

Corresponding author(s): Laurence Pelletier

Last updated by author(s): 7/31/2022

Reporting Summary

Nature Research wishes to improve the reproducibility of the work that we publish. This form provides structure for consistency and transparency in reporting. For further information on Nature Research policies, see our Editorial Policies and the Editorial Policy Checklist.

Statistics

For all statistical analyses, confirm that the following items are present in the figure legend, table legend, main text, or Methods section.

n/a	Confirmed	
☐	☒	The exact sample size (n) for each experimental group/condition, given as a discrete number and unit of measurement
☐	☒	A statement on whether measurements were taken from distinct samples or whether the same sample was measured repeatedly
☒	☐	The statistical test(s) used AND whether they are one- or two-sided<br>*Only common tests should be described solely by name; describe more complex techniques in the Methods section.*
☒	☐	A description of all covariates tested
☒	☐	A description of any assumptions or corrections, such as tests of normality and adjustment for multiple comparisons
☒	☐	A full description of the statistical parameters including central tendency (e.g. means) or other basic estimates (e.g. regression coefficient) AND variation (e.g. standard deviation) or associated estimates of uncertainty (e.g. confidence intervals)
☒	☐	For null hypothesis testing, the test statistic (e.g. F, t, r) with confidence intervals, effect sizes, degrees of freedom and P value noted<br>*Give P values as exact values whenever suitable.*
☒	☐	For Bayesian analysis, information on the choice of priors and Markov chain Monte Carlo settings
☒	☐	For hierarchical and complex designs, identification of the appropriate level for tests and full reporting of outcomes
☒	☐	Estimates of effect sizes (e.g. Cohen's d, Pearson's r), indicating how they were calculated

Our web collection on statistics for biologists contains articles on many of the points above.

Software and code

Policy information about availability of computer code

Data collection	To acquire images we used NIS elements (https://www.microscope.healthcare.nikon.com/products/software/nis-elements). To analyze, crop and LUT images we used ImageJ (https://imagej.nih.gov/ij/).
Data analysis	Our own denoiser along with all the methods to which we compared ourselves, our script for calculating PSNR/SSIM, and our script for adding synthetic Gaussian noise, and all benchmarking datasets are included on GitHub: https://github.com/pelletierlab/noise2fast. We also use CellPose for segmentation which is available here: https://github.com/MouseLand/cellpose.

For manuscripts utilizing custom algorithms or software that are central to the research but not yet described in published literature, software must be made available to editors and reviewers. We strongly encourage code deposition in a community repository (e.g. GitHub). See the Nature Research guidelines for submitting code & software for further information.

Data

Policy information about availability of data

All manuscripts must include a data availability statement. This statement should provide the following information, where applicable:

- Accession codes, unique identifiers, or web links for publicly available datasets
- A list of figures that have associated raw data
- A description of any restrictions on data availability

We have made use of BSD68 dataset, available here: https://www2.eecs.berkeley.edu/Research/Projects/CS/vision/bsds/, Set12 which is available here: https://paperswithcode.com/dataset/set12 and the Fluorescence Microscopy Dataset (FMD) available here: https://curate.nd.edu/show/f4752f78z6t. Benchmarking datasets along with code and reproducibility instructions for Figure 2 are available on our GitHub (https://github.com/pelletierlab/Noise2Fast). Note that all speed benchmarks were performed on an RTX 5000 mobile GPU, and therefore results may vary according to GPU used. Source input and output images used to make the graphs in Figure 4 are publicly available on our GitHub (Noise2Fast/Fig5Data). Source data for Figure 3 is available on our GitHub as well (Noise2Fast/livecells).

Figure 1 is a conceptual illustration and does not make use of any datasets, however the image we use to illustrate checkerboard downsampling is a crop of an image available on our GitHub (Noise2Fast/BSD68/19.tif). The minimum dataset for the experiment illustrated in Figure 5 is publicly available on our GitHub (Noise2Fast/Fig6Data).

Field-specific reporting

Please select the one below that is the best fit for your research. If you are not sure, read the appropriate sections before making your selection.

☒ Life sciences ☐ Behavioural & social sciences ☐ Ecological, evolutionary & environmental sciences

For a reference copy of the document with all sections, see nature.com/documents/nr-reporting-summary-flat.pdf

Life sciences study design

All studies must disclose on these points even when the disclosure is negative.

Sample size	Sample sizes were selected to maximize comparability to other methods. For example, the BSD68 dataset contains 68 images and is widely used for benchmarking Gaussian denoisers, the same is true of Set12. For FMD we selected 5 diverse images because of the massive computational resources required to run some of our compared methods to completion on a 512x512 image.
Data exclusions	For FMD we selected 5 representative images to use as benchmark due to computational limitations, however this selection was done at the outset before any testing was done and our results on this dataset agree with our results on other datasets, at least in terms of how each method ranks.
Replication	We have made our code publicly available with full reproducibility instructions. The time, technical skill, and computational resources required to reproduce our results are minimal.
Randomization	Synthetic Gaussian noise was randomly generated using pythons build in random number generator.
Blinding	Blinding is not applicable to this study.

Reporting for specific materials, systems and methods

We require information from authors about some types of materials, experimental systems and methods used in many studies. Here, indicate whether each material, system or method listed is relevant to your study. If you are not sure if a list item applies to your research, read the appropriate section before selecting a response.

Materials & experimental systems

n/a	Involved in the study	
☒	☐	Antibodies
☐	☒	Eukaryotic cell lines
☒	☐	Palaeontology and archaeology
☒	☐	Animals and other organisms
☒	☐	Human research participants
☒	☐	Clinical data
☒	☐	Dual use research of concern

Methods

n/a	Involved in the study	
☒	☐	ChIP-seq
☒	☐	Flow cytometry
☒	☐	MRI-based neuroimaging

Eukaryotic cell lines

Policy information about cell lines

Cell line source(s)	MDA-MB 231 cell line was provided by Dr. Robert S. Kerbel (Sunnybrook Health Sciences Centre, Toronto, Canada) and RPE-1 cell line was purchased from American Type Culture Collection (ATCC)
Authentication	None of the cell lines were authenticated
Mycoplasma contamination	MDA-MB 231 and RPE-1 cell lines were negative for mycoplasma
Commonly misidentified lines (See ICLAC register)	*Name any commonly misidentified cell lines used in the study and provide a rationale for their use.*
